# Access to care through telehealth among U.S. Medicare beneficiaries in the wake of the COVID-19 pandemic

**DOI:** 10.3389/fpubh.2022.946944

**Published:** 2022-09-06

**Authors:** Min Lu, Xinyi Liao

**Affiliations:** Department of Public Health Sciences, Miller School of Medicine, University of Miami, Miami, FL, United States

**Keywords:** COVID-19, telehealth, access to care, Medicare, random forests

## Abstract

**Background:**

The coronavirus disease 2019 (COVID-19) public health emergency has amplified the potential value of deploying telehealth solutions. Less is known about how trends in access to care through telehealth changed over time.

**Objectives:**

To investigate trends in forgone care and telehealth coverage among Medicare beneficiaries during the COVID-19 pandemic.

**Methods:**

A cross-sectional study design was used to analyze the outcomes of 31,907 Medicare beneficiaries using data from three waves of survey data from the Medicare Current Beneficiary Survey COVID-19 Supplement (Summer 2020, Fall 2020, and Winter 2021). We identified informative variables through a multivariate classification analysis utilizing Random Forest machine learning techniques.

**Findings:**

The rate of reported forgone medical care because of COVID-19 decreased largely (22.89–3.31%) with a small increase in telehealth coverage (56.24–61.84%) from the week of June 7, 2020, to the week of April 4 to 25, 2021. Overall, there were 21.97% of respondents did not know whether their primary care providers offered telehealth services; the rates of forgone care and telehealth coverage were 11.68 and 59.52% (11.73 and 81.18% from yes and no responses). Our machine learning model predicted the outcomes accurately utilizing 43 variables. Informative factors included Medicare beneficiaries' age, Medicare-Medicaid dual eligibility, ability to access basic needs, certain mental and physical health conditions, and interview date.

**Conclusions:**

This cross-sectional survey study found proliferation and utilization of telehealth services in certain subgroups during the COVID-19 pandemic, providing important access to care. There is a need to confront traditional barriers to the proliferation of telehealth. Policymakers must continue to identify effective means of maintaining continuity of care and growth of telehealth services.

## 1. Introduction

Access to medical care is an ongoing problem for vulnerable populations (1, 2), and the COVID-19 pandemic has had a devastating impact on disparities in forgone care (3–5). There were about 40% of U.S. adults reporting forgone medical care during the COVID-19 pandemic, citing fear of infection among the reasons (6). Another reason is that the physician's office may present logistical barriers, such as inconvenient clinic hours and lack of care coordinators (7). In the meanwhile, telehealth virtual visits offer a way to reduce exposure to COVID-19 infections. When Medicare beneficiaries tend to reduce traditional in-person medical visits, telehealth has been widely utilized because of its usability and safety in providing healthcare services (8, 9).

Several studies that contributed to the use of telehealth were published during the initial stage of the COVID-19 pandemic when health systems lacked medical supplies and staff (9–13). However, no telehealth program can be created overnight. There has been limited investigation since telemedical innovations and vaccine administration were implemented (14–20). After health systems regained the capacity to treat patients in person and the diverse contributions of telehealth were made, there is limited understanding of the experiences of Medicare beneficiaries, who are a higher risk population for COVID-19 mortality since most of them are 65 years or older (4).

In this study, we examined trends in patient-reported access to care and telehealth utilization among Medicare beneficiaries in three waves of data collection during the COVID-19 pandemic (Summer 2020, Fall 2020, and Winter 2021). We expected that these two outcomes were correlated, and therefore we conducted a multivariate classification analysis. Reasons for disparities included socio-demographic factors, personal experiences with COVID-19, electronic device usage, economic and mental effects of the pandemic, non-COVID-19 health status and interview date. Since there are many correlated predictors with missing values, multivariate classification analysis utilized Random Forest machine learning techniques (21–23).

## 2. Methods

### 2.1. Data

Data sets were downloaded from the Medicare Current Beneficiary Survey (MCBS) COVID-19 Supplement Public Use File, collected *via* a telephone survey in Summer 2020 (June to July), Fall 2020 (October to November), and Winter 2021 (February to April). These three waves of survey data contained a nationally representative sample of all Medicare beneficiaries, and the survey was conducted in either English or Spanish. The MCBS is sponsored by the Centers for Medicare & Medicaid Services (CMS) in the U.S., and the original MCBS primarily has focused on economic and beneficiary topics, including health care use, access barriers, and expenditures. With the emergence of COVID-19, CMS was uniquely positioned to collect vital information on how the pandemic is impacting the Medicare population by using the MCBS as a vehicle to collect data. Ethics approval and consent to participate in the entire project were obtained by CMS and NORC at the University of Chicago; both organizations uphold provisions established under the Privacy Act of 1974, the NORC Institutional Review Board (IRB), the Office of Management and Budget, and the Federal Information Security Management Act of 2002.

In total, 235 variables were included in all three waves of surveys (see Supplementary Table 1). Among the three variables describing interview characteristics, interview week was included as a predictor since it is more relevant than the other two variables, interview language and interview with proxy. We utilized all the variables recording beneficiaries' demographic information as predictors. Among the 121 variables describing access to care during the pandemic, two variables were chosen as primary outcomes: forgone medical care because of COVID-19 and the status of whether primary care physicians (PCP) offered telehealth appointments by the date of interview. The description of these two outcome variables in the MCBS survey is listed in Supplementary Table 2. We chose five predictors from these 121 variables since they are relevant to telehealth, including owning a computer, owning a smartphone, owning a tablet, access to the Internet, and using video/voice calls; other variables in this group are too sparse to be added in the classification model since most of them are follow-up questions if beneficiaries forwent medical care, such as unable to get care for vision, dental, hearing, etc. Most of the beneficiaries did not have experience with COVID-19. From the 42 variables describing beneficiaries' personal experiences with COVID-19 and 27 variables describing preventive measures and knowledge about COVID-19, we chose two variables, including the results for COVID-19 and COVID-19 antibody tests—they were included in the descriptive summary but not included in the classification model because of missing data since most respondents did not conduct the tests. We included all the variables describing the economic and mental effects of the pandemicӑand beneficiaries' non-COVID-19 health status.

### 2.2. Statistical analysis

To investigate patterns of access to care and telehealth offerings among Medicare beneficiaries during COVID-19, we first run descriptive analyses for 46 independent variables, including socio-demographic factors, personal experiences with COVID-19, electronic device usage, etc. Next, we conducted a multivariate classification analysis to assess whether these variables were significant predictors of access to care and telehealth utilization. In this step, a variable recording interview date was added, while four variables were excluded because they are only available in the survey conducted in Winter 2021, including two socio-demographic variables (Medicare Advantage and Part D plan) and two variables describing personal experiences with COVID-19 (COVID-19 test and COVID-19 antibody test results).

For all analyses, a complex sample design was used with sampling weights provided by the MCBS to produce nationally representative estimates. All percentages and proportions that appeared in this study were calculated using survey weights. In the descriptive analyses, weighted chi-squared tests were used to test the association between each predictor and the outcome. In the multivariate classification analysis, Random Forest (21) model was applied for the prediction of outcomes, a modern machine learning technique that has been utilized widely to explore a large number of predictors and identify replicable sets of risk factors (24–28). Weighted chi-squared tests and the Random Forest model were implemented in the open-source R software using the weights (29) and randomForestSRC (23, 30) packages correspondingly. From the randomForestSRC package, the functions rfsrc and tune were applied with 1,000 trees. The parameters case.wt and na.action were set for survey weighting and missing data imputation for independent variables. All statistical inferences were based on a significance level of *P* (two-sided) ≤ 0.05. Model performance was evaluated through out-of-bag misclassification error, where out-of-bag refers to the data proportion that is not used for fitting the model (Classification trees were “grown” from bootstrap samples of the original dataset, leaving an average of 37% of unsampled data referred to as out-of-bag data) but for calculating the cross-validated prediction error and VIMP.

#### 2.2.1. Variable importance (VIMP) and partial plot

From the Random Forest classification model, the estimated VIMP (21, 22) was adopted for ranking variables, which utilizes a prediction-based approach by estimating classification error attributable to the predictor. The VIMP can be interpreted as the increase in the misclassification error when the corresponding predictor is randomly permutated into a noise variable. For example, a VIMP of 4.29% indicates that a variable improves by 4.29% the ability of the model to classify the status of the outcome. Standard errors and *P*-values were generated by a delete-*d*-jackknife procedure (22, 31). In addition, partial dependence plots were used to visualize the variables' impact on the outcome through mapping their marginal effects (32, 33), where predicted probability is adjusted by integrating out all variables other than the selected variable. Inferences of VIMP and partial plots were generated using the functions subsample and plot.variable (setting partial = TRUE) from the randomForestSRC R package with default settings.

## 3. Results

### 3.1. Description of the sample

**Figure 1:**
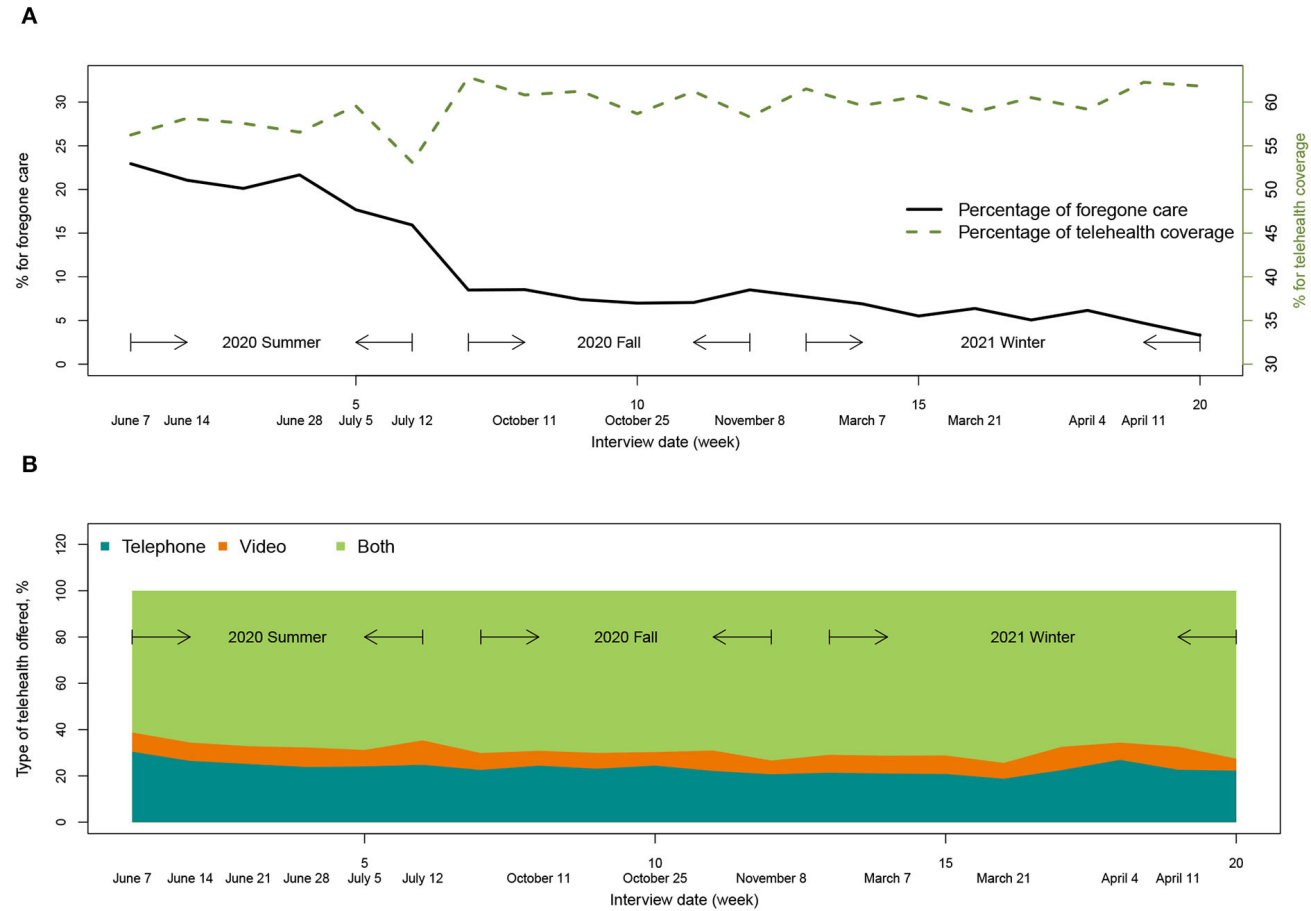
Trends of forgone care and telehealth utilization. **(A)** The percentage of forgone care decreased largely with a small increase in telehealth coverage. **(B)** The usage of both video and telephone for telehealth increased over time.

The main characteristics of the sample from the three waves of survey data are displayed in Table 1. There are 46 variables in the rows, including ten socio-demographic variables, two variables describing personal experiences with COVID-19, five variables describing electronic device usage, seven variables describing economic and mental effects of the pandemic, and 22 variables recording non-COVID-19 health status. Among these groups of variables, the two variables describing personal experiences with COVID-19, which recorded COVID-19 test and COVID-19 antibody test results, are not significantly associated with any of the two outcomes; all the five variables describing electronic device usage are significantly associated with both outcomes. The variable recording interview date is displayed in Figure 1 plotted against the percentage of forgone care and type of telehealth provided by PCP. From Summer 2020 to Winter 2021, the proportion of forgone care decreased largely from 22.89 to 3.31%. However, the increase in telehealth coverage is not as large (56.24 to 61.84%), as shown in Figure 1. The type of telehealth offered was summarized as “telephone,” “video,” and “both,” whose survey-weighted percentages in June 2020 were 30.46, 8.30, and 61.24%, respectively, and in April 2021 were 22.30, 5.12, and 72.58%, respectively. There was an increase in the usage of both video and telephone for telehealth.

**Table 1:** Descriptive analysis of forgone care and telehealth coverage reported by Medicare beneficiaries.

		**Number (Survey-weighted percentage** ^**†**^ **)**					
			**Unable to get care because of COVID-19**		**Primary care physician (PCP) offers telehealth**		
**Variable**	**Category**	**Frequency**	**Yes**	**No**	**Yes**	**No**	**Sig^‡^**
Overall	Overall	31,907	3,556 (12)	28,209 (88)	18,248 (81)	4,996 (19)	
Age	0–65	5,993 (17)	803 (13)	5,148 (87)	3,351 (79)	995 (21)	***+++
	65–74	11,032 (51)	1,365 (13)	9,629 (87)	6,828 (85)	1,323 (15)	
	74+	14,882 (33)	1,388 (10)	13,432 (90)	8,069 (77)	2,678 (23)	
Gender	Male	14,378 (45)	1,507 (11)	12,813 (89)	8,092 (80)	2,308 (20)	***++
	Female	17,529 (55)	2,049 (12)	15,396 (88)	10,156 (82)	2,688 (18)	
Race/ethnicity	White non-hispanic	23,808 (76)	2,783 (12)	20,931 (88)	13,676 (83)	3,325 (17)	***+++
	Black non-hispanic	3,138 (10)	257 (9)	2,864 (91)	1,633 (71)	747 (29)	
	Hispanic	3,246 (8)	332 (11)	2,894 (89)	1,955 (77)	615 (23)	
	Other/Unknown	1,715 (6)	184 (11)	1,520 (89)	984 (79)	309 (21)	
Metro residence	Metro	24,381 (80)	2,774 (12)	21,503 (88)	14,705 (83)	3,445 (17)	+++
	Non-metro	7,510 (20)	780 (11)	6,692 (89)	3,533 (73)	1,547 (27)	
Region	Northeast	5,617 (18)	705 (13)	4,887 (87)	3,360 (82)	811 (18)	***+++
	Midwest	7,241 (22)	883 (12)	6,333 (88)	4,039 (83)	1,001 (17)	
	South	12,421 (39)	1,093 (10)	11,268 (90)	6,587 (77)	2,380 (23)	
	West	6,617 (22)	874 (14)	5,711 (86)	4,257 (86)	800 (14)	
Income	<$25,000	11,649 (31)	1,169 (10)	10,410 (90)	5,984 (73)	2,352 (27)	***+++
	$25,000 or more	18,891 (69)	2,277 (13)	16,556 (87)	11,602 (85)	2,362 (15)	
Non-English	Yes	3,928 (11)	405 (10)	3,496 (90)	2,335 (77)	739 (23)	**+++
	No	27,948 (89)	3,148 (12)	24,685 (88)	15,900 (82)	4,246 (18)	
Medicare-Medicaid	Full	4446 (10)	481 (11)	3,932 (89)	2,398 (73)	855 (27)	+++
dual eligibility	Nondual	25,298 (85)	2,841 (12)	22,356 (88)	14,748 (83)	3,642 (17)	
	Partial	1,116 (3)	123 (11)	991 (89)	562 (72)	243 (28)	
	QMB only	1,047 (3)	111 (10)	930 (90)	540 (70)	256 (30)	
Medicare advantage	No MA enrollment	11,606 (59)	801 (7)	10,744 (93)	6617 (82)	1777 (18)	
(MA)	Partial-year MA	517 (4)	47 (9)	467 (91)	296 (83)	69 (17)	
	Full-year MA	8,661 (37)	566 (7)	8,060 (93)	5,215 (81)	1,362 (19)	
Part D plan	Yes	8,991 (78)	539 (7)	8,411 (93)	5,228 (80)	1,508 (20)	
	No	2,112 (22)	141 (7)	1,958 (93)	1,260 (82)	307 (18)	
Positive COVID-19 test	Yes	571 (9)	50 (9)	515 (91)	371 (85)	77 (15)	
	No	5,183 (89)	430 (9)	4,732 (91)	3,282 (83)	775 (17)	
	No results yet	88 (2)	10 (14)	77 (86)	59 (80)	12 (20)	
Positive COVID-19	Yes	104 (15)	14 (14)	90 (86)	72 (86)	15 (14)	
antibody test	No	508 (83)	49 (9)	455 (91)	349 (86)	62 (14)	
	No results yet	17 (3)	0 (0)	17 (100)	10 (94)	1 (6)	
Own computer	Yes	18,952 (65)	2,398 (13)	16,489 (87)	11,860 (86)	2,254 (14)	***+++
	No	12,867 (35)	1,153 (9)	11,642 (91)	6,344 (71)	2,727 (29)	
Own smartphone	Yes	19,976 (70)	2,526 (13)	17,372 (87)	12,473 (85)	2,523 (15)	***+++
	No	11,573 (30)	1,016 (9)	10,504 (91)	5,624 (71)	2,409 (29)	
Own tablet	Yes	12,723 (45)	1,669 (14)	11,012 (86)	8,217 (87)	1,416 (13)	***+++
	No	19,113 (55)	1,879 (10)	17,139 (90)	10,001 (76)	3,572 (24)	
Access to internet	Yes	25,024 (84)	3,056 (13)	21,875 (87)	15,326 (84)	3,270 (16)	***+++
	No	6,724 (16)	490 (7)	6,192 (93)	2,856 (64)	1,702 (36)	
Use video/voice calls	Yes	13,836 (48)	2,049 (15)	11,740 (85)	9,248 (88)	1,409 (12)	***+++
	No	17,926 (52)	1,490 (9)	16,350 (91)	8,939 (74)	3,566 (26)	
Able to pay rent or	Able	18,799 (61)	2,133 (12)	16,587 (88)	10,955 (81)	2,966 (19)	***
mortgage	Unable	510 (2)	91 (18)	412 (82)	291 (78)	97 (22)	
	Not needed	12,474 (37)	1,318 (12)	11,109 (88)	6,946 (81)	1,914 (19)	
Able to get food	Able	30,338 (95)	3,253 (11)	26,966 (89)	17,401 (81)	4,729 (19)	***++
	Unable	1,011 (3)	246 (25)	753 (75)	562 (77)	173 (23)	
	Not needed	488 (1)	50 (13)	433 (87)	257 (78)	80 (22)	
Able to get home	Able	28,950 (91)	2,945 (11)	25,887 (89)	16,595 (81)	4,554 (19)	***
supplies	Unable	2,000 (7)	490 (26)	1,498 (74)	1,174 (82)	297 (18)	
	Not needed	883 (2)	111 (13)	767 (87)	447 (79)	129 (21)	
Feel financially	More secure	1,198 (4)	131 (12)	1,064 (88)	714 (84)	166 (16)	***
secure	Less secure	4,038 (15)	793 (20)	3,222 (80)	2,363 (81)	647 (19)	
	About the same	22,478 (80)	2,230 (10)	20,164 (90)	12,838 (82)	3,380 (18)	
Feel stressed	More stressed	10,833 (42)	1,793 (17)	8,992 (83)	6,759 (84)	1,420 (16)	***+++
	Less stressed	925 (3)	70 (9)	851 (91)	508 (78)	172 (22)	
	About the same	15,950 (55)	1,298 (8)	14,597 (92)	8,633 (80)	2,615 (20)	
Feel lonely or sad	More lonely or sad	5,939 (22)	1,045 (18)	4,859 (82)	3,623 (83)	845 (17)	***++
	Less lonely or sad	920 (3)	91 (11)	825 (89)	527 (80)	146 (20)	
	About the same	20,810 (75)	2,012 (10)	18,726 (90)	11,726 (81)	3,205 (19)	
Feel socially	More connected	2,840 (10)	360 (13)	2,468 (87)	1,710 (82)	428 (18)	***+++
connected	Less connected	10,116 (38)	1,512 (15)	8,567 (85)	6,149 (84)	1,348 (16)	
	About the same	14,777 (51)	1289 (9)	13,429 (91)	8050 (80)	2,426 (20)	
Weak immune	Yes	5,464 (18)	933 (18)	4,504 (82)	3,581 (85)	724 (15)	***+++
system	No	26,294 (82)	2,606 (10)	23,579 (90)	14,589 (80)	4,254 (20)	
Hypertension or high	Yes	20,416 (63)	2,238 (12)	18,089 (88)	11,921 (80)	3,400 (20)	+++
BP	No	11,422 (37)	1,308 (12)	10,061 (88)	6,290 (83)	1,581 (17)	
Myocardial infarction	Yes	3,226 (10)	343 (11)	2,864 (89)	1,854 (79)	568 (21)	++
	No	28,575 (90)	3,196 (12)	25,258 (88)	16,334 (81)	4,408 (19)	
Angina pectoris/CHD	Yes	2,750 (8)	374 (15)	2,364 (85)	1,645 (81)	450 (19)	***
	No	28,943 (92)	3,160 (11)	25,653 (89)	16,495 (81)	4,500 (19)	
Congestive heart	Yes	2,056 (6)	252 (13)	1,795 (87)	1,189 (77)	396 (23)	+++
failure	No	29,736 (94)	3291 (12)	26,312 (88)	16,997 (81)	4,581 (19)	
Other heart condition,	Yes	7,408 (22)	954 (13)	6,419 (87)	4,378 (80)	1,218 (20)	***+
e.g., valve/rhythm	No	24,292 (78)	2,562 (11)	21,624 (89)	13,766 (82)	3,736 (18)	
Stroke/brain	Yes	3,256 (9)	375 (12)	2,865 (88)	1,865 (78)	579 (22)	+++
hemorrhage	No	28,575 (91)	3,170 (12)	25,279 (88)	16,339 (82)	4,404 (18)	
High cholesterol	Yes	20,394 (64)	2281 (12)	18,026 (88)	11,983 (81)	3,278 (19)	*
	No	11,371 (36)	1,259 (11)	10,058 (89)	6,186 (81)	1,694 (19)	
Cancer (non-skin)	Yes	6,342 (19)	784 (13)	5,530 (87)	3,797 (82)	990 (18)	***
	No	25,504 (81)	2,765 (11)	22,626 (89)	14,420 (81)	3,994 (19)	
Alzheimers/dementia	Yes	1,280 (3)	126 (11)	1,146 (89)	723 (76)	249 (24)	+++
	No	30,582 (97)	3,424 (12)	27,024 (88)	17,504 (81)	4,740 (19)	
Depression	Yes	8,523 (27)	1,221 (15)	7,249 (85)	5,174 (82)	1,349 (18)	***
	No	23,289 (73)	2,320 (11)	20,881 (89)	13,016 (81)	3,633 (19)	
Osteoporosis or soft	Yes	5,975 (18)	797 (15)	5,150 (85)	3,653 (82)	887 (18)	***+
bones	No	25,778 (82)	2,743 (11)	22,922 (89)	14,509 (81)	4,083 (19)	
Broken hip	Yes	1,196 (3)	133 (12)	1,061 (88)	669 (79)	205 (21)	
	No	30,659 (97)	3,419 (12)	27,101 (88)	17,549 (81)	4,781 (19)	
Emphysema/asthma/	Yes	6,180 (19)	866 (15)	5,286 (85)	3,756 (81)	997 (19)	***
COPD	No	25,661 (81)	2,682 (11)	22,866 (89)	14,456 (81)	3,988 (19)	
Diabetes/high blood	Yes	10,175 (33)	1,270 (13)	8,851 (87)	6,196 (81)	1,662 (19)	***
sugar	No	21,659 (67)	2,278 (11)	19,293 (89)	12,022 (81)	3,316 (19)	
Any arthritis	Yes	11,436 (61)	1,459 (13)	9,924 (87)	6791 (80)	1,884 (20)	***
	No	7,424 (39)	708 (10)	6,685 (90)	4,008 (80)	1,176 (20)	
Any heart condition	Yes	10,589 (32)	1,292 (13)	9,247 (87)	6,184 (80)	1,777 (20)	***+++
	No	20,585 (68)	2,154 (11)	18,343 (89)	11,635 (82)	3,093 (18)	
Any osteoporosis	Yes	6,544 (20)	838 (14)	5,677 (86)	3,952 (82)	979 (18)	***
or broken hip	No	24,621 (80)	2,617 (11)	21,895 (89)	13,861 (81)	3,888 (19)	
Ever smoke cigarette	Yes	17,552 (58)	1,984 (12)	15,488 (88)	10,061 (81)	2,734 (19)	
/cigar/pipe	No	13,718 (42)	1,478 (11)	12,181 (89)	7,812 (82)	2,149 (18)	
Currently smoke	Yes	3,412 (21)	399 (11)	2,990 (89)	1,848 (78)	597 (22)	+++
cigarette/cigar/pipe	No	14,125 (79)	1,585 (12)	12,486 (88)	8,205 (82)	2,135 (18)	
Ever used e-cigarette	Yes	2,745 (9)	389 (14)	2,334 (86)	1,539 (80)	428 (20)	***
	No	28,477 (91)	3,071 (11)	25,293 (89)	16,319 (81)	4,443 (19)	
Smoke e-cigarette now	Yes	377 (15)	54 (15)	321 (85)	211 (78)	63 (22)	
	No	2,362 (85)	335 (14)	2,007 (86)	1,328 (81)	362 (19)	

† Categories of “inapplicable/missing,” “don't know,” “not ascertained,” and “refused” were excluded in calculating percentages and weighted chi-squared statistics.

‡ Sig indicates significant level according to *P*-values: when the outcome is forgone care, *p ≤ 0.05, **p ≤ 0.01, ***p ≤ 0.001; when the outcome is telehealth coverage, + *p* ≤ 0.05, ++ *p* ≤ 0.01, and +++ *p* ≤ 0.001.

In total, there are 31,907 Medicare beneficiaries included in the final sample, among which 11,114 are from Summer 2020, 9,686 from Fall 2020 and 11,107 from Winter 2021. For the two outcomes, 135 and seven beneficiaries reported “don't know” and “refused,” respectively, for answering whether they were unable to get care because of COVID-19; 7,174 and three beneficiaries reported “don't know” and “refused,” respectively, with 1,486 inapplicable/missing data for answering whether PCP offered telehealth appointments. These categories were discarded in the descriptive analysis for both outcomes and independent variables shown in Table 1. There were 21.97% of respondents unknown whether their PCP offered telehealth services; the rates of forgone care and telehealth coverage were 11.68 and 59.52% (11.73 and 81.18% from yes and no responses). Forgone care was negatively correlated telehealth coverage (χ^2^ = 18.40, *p* < 0.001).

Among the 10 socio-demographic variables, six were significantly associated with both outcomes including age, gender, race/ethnicity, region, income and use of a language other than English at home (shown as non-English in Table 1). Beneficiaries who were metro residents and not eligible for Medicaid benefits (non-dual-eligible) significantly tended to have telehealth coverage. Among the seven variables describing economic and mental effects of the pandemic, four were significantly associated with both outcomes, indicating that beneficiaries who were able to get food and felt less stressed, lonely, or sad and more socially connected were more likely to have access to care; beneficiaries with access to care were those who were able to or need not pay rent/mortgage as well as get home supplies and those who did not feel less financially secure. Most of the 22 variables recording non-COVID-19 health status were significantly associated with either of the outcomes.

### 3.2. Relationship of outcomes to important variables

We selected variables for predicting both outcomes by machine learning using the Random Forest multivariate classification model shown in Table 2. Only yes and no responses of the outcomes were included in this classification model (*n* = 22, 138, *p* = 43). The Random Forest model predicts the outcomes accurately: the out-of-bag misclassification error is 11.63% for predicting forgone care and 21.18% for telehealth coverage. The full version of Table 2 can be found in Supplementary Table 3. For method comparison, we also analyzed the data with logistic regression. The model of regular logistic regression did not converge because of the missing data problem. Utilizing penalized logistic regression (34, 35) with 10-fold cross-validation provides misclassification errors of 11.94% for predicting forgone care and 21.91% for telehealth coverage; the coefficients were listed in Supplementary Table 4.

**Table 2:** Informative variables from multivariate classification analysis using Random Forest.

	**Unable to get care**				**Primary care physician**				
	**because of COVID-19**				**(PCP) offers telehealth**				
**Variable**	**Est**	**SE**	***P*-value**	**OR^§^**	**Est**	**SE**	***P*-value**	**OR**	**Sig^‡^**
Age	0.13	0.04	0.002	1.08	0.49	0.10	0.000	0.68	**+++
Medicare-medicaid dual eligibility	0.50	0.22	0.010	0.92	4.81	0.59	0.000	0.58	**+++
Use video/voice calls	0.09	0.04	0.008	1.86	0.34	0.10	0.000	2.60	**+++
Able to pay rent/mortgage	0.41	0.18	0.013	0.61	1.40	0.35	0.000	1.20	*+++
Able to get food	0.90	0.40	0.012	0.39	2.17	0.45	0.000	1.32	*+++
Able to get home supplies	1.20	0.33	0.000	0.34	0.59	0.36	0.049	0.98	***+
Feel financially secure	0.71	0.15	0.000	0.56	1.49	0.27	0.000	1.21	***+++
Feel lonely or sad	0.33	0.16	0.017	1.71	1.39	0.30	0.000	1.28	*+++
Angina pectoris/CHD	0.23	0.09	0.005	1.38	0.63	0.16	0.000	0.98	**+++
Congestive heart failure	0.29	0.08	0.000	1.14	1.18	0.18	0.000	0.78	***+++
Other heart cond, eg valve/rhythm	0.04	0.02	0.035	1.22	0.29	0.07	0.000	0.91	*+++
Stroke/brain hemorrhage	0.29	0.06	0.000	1.07	0.50	0.16	0.001	0.81	***++
Cancer (non-skin)	0.06	0.03	0.031	1.16	0.42	0.08	0.000	1.06	*+++
Alzheimers/dementia	0.48	0.09	0.000	0.89	1.18	0.25	0.000	0.71	***+++
Depression	0.08	0.03	0.005	1.50	0.31	0.06	0.000	1.04	**+++
Osteoporosis/soft bones	0.10	0.03	0.000	1.37	0.19	0.06	0.000	1.09	***+++
Broken hip	0.22	0.12	0.037	1.00	0.89	0.31	0.002	0.86	*++
Emphysema/asthma/COPD	0.16	0.03	0.000	1.42	0.14	0.08	0.034	1.00	***+
Ever used e-cigarette	0.15	0.07	0.015	1.24	0.63	0.25	0.005	0.92	*++
Interview date	2.09	0.27	0.000	–	1.16	0.42	0.003	–	***++

Est and SE indicate estimation and standard error for Random Forest variable importance (VIMP).

§ OR indicates survey-weighted odds ratio indicating the direction of effects: if the value is larger than one, the first category of the variable in Table 1 is more likely with positive outcome than the second category. For example, the odds ratio of age is 1.08, indicating that the 0 to 65 age group was more likely with telehealth coverage than the 65–74 age group.

‡ Sig indicates significant level according to *P* values of VIMP: when the outcome is forgone care, *p ≤ 0.05, **p ≤ 0.01, ***p ≤ 0.001; when the outcome is telehealth coverage, + *p* ≤ 0.05, ++ *p* ≤ 0.01, and +++ *p* ≤ 0.001.

Table 2 presents the estimate, standard error (SE), and *P*-value of Random Forest VIMP. A large estimate of VIMP indicates a variable that is more informative for predicting the corresponding outcome, while a negative estimate indicates a noise variable. For example, a VIMP value of 4.81% for forgone care indicates that the variable improves by 4.81% the ability of the model to classify the status of forgone care. However, VIMP can not provide the direction of the association, for which we used the odds ratio (OR). To interpret the OR conveniently, we can consider Table 1 as a list of stacked contingency tables of variables, and we used the first two rows of each contingency table for each variable for calculating an OR with survey weights. Odds ratios >1 indicate a positive association between the first category of the variable of interest and the corresponding outcome compared with its second category; odds ratios <1 represent a negative association. For binary variables with yes or no response, odds ratios >1 indicate a positive association since the first category is always for the yes response.

**Figure 2:**
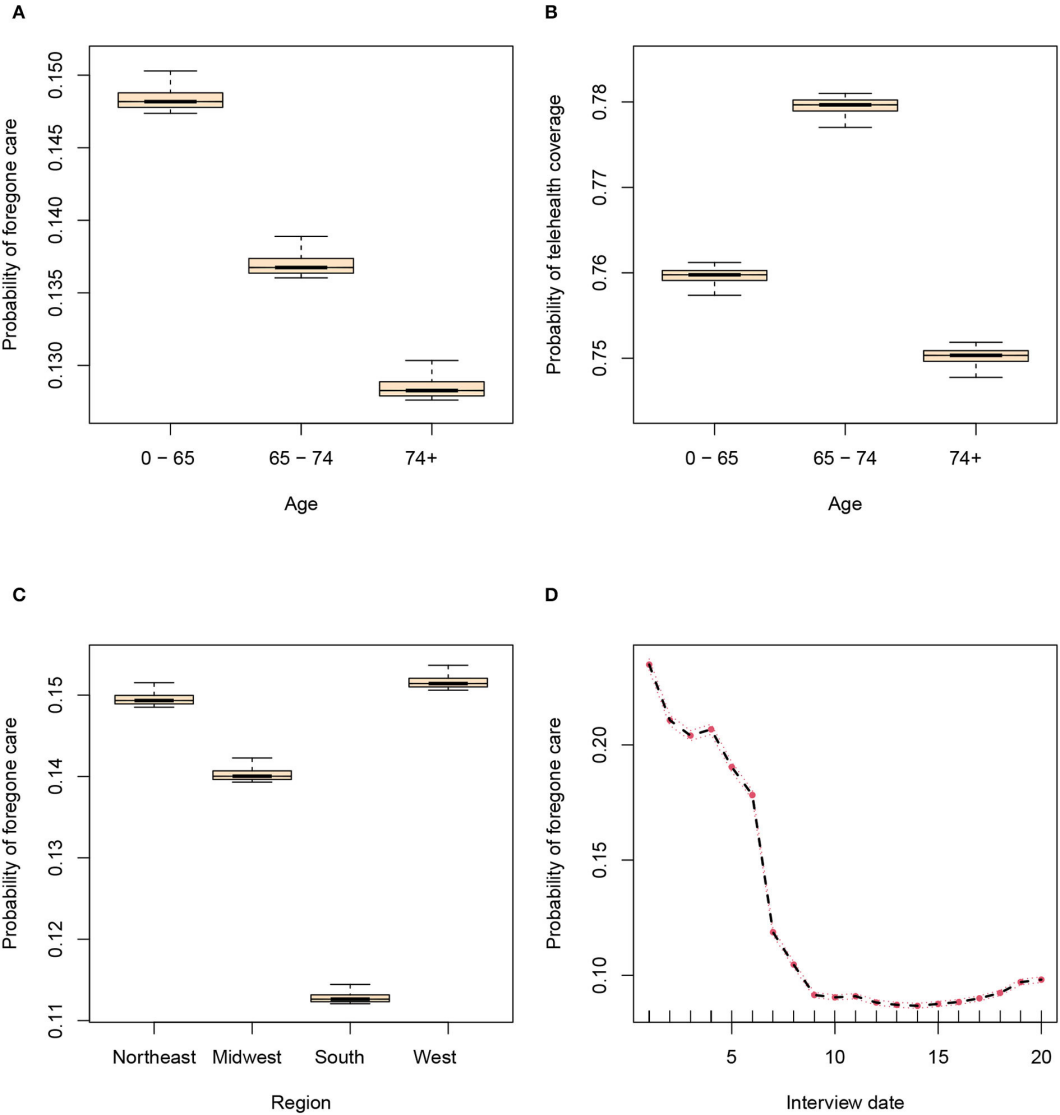
Random forest estimated probabilities of outcomes plotted against candidate variables after adjusting for other variables. **(A)** The association between age and forgone care. **(B)** The association between age and telehealth coverage. **(C)** The association between region and forgone care. **(D)** The association between interview date and forgone care.

We detected 20 variables that were significantly associated with both forgone care and telehealth coverage, as shown in Table 2. Two variables are not significantly associated with any of the two outcomes, statuses of owning a tablet and having any heart condition (see Supplementary Table 3). However, variables describing specific heart conditions are significantly associated with the outcomes, which possibly masks the effect of having any heart condition as the overall status. The effects of age, census region, and race/ethnicity are shown in Figure 2 and Supplementary Figure 1. As demonstrated in Figure 2D, the probability of forgone care decreased largely across time after adjusting for other variables, indicating a strengthened health system.

#### 3.2.1. Forgone care

The most informative variable is interview date for predicting if the beneficiary was unable to get care because of COVID-19, contributing 2.09% prediction accuracy (SE = 0.27, *p* < 0.001). The status of being able to get home supplies is the second most informative variable, contributing 1.20% prediction accuracy (SE = 0.33, *p* < 0.001). Medicare-Medicaid dual eligibility and age are also significantly associated with the outcome, indicating that non-dual-eligible beneficiaries (not eligible for Medicaid benefits) with younger age were more likely to forgo care. People who reported using video/voice calls were more likely to forgo care. The groups that reported being unable to pay rent/mortgage or get food or home supplies were more likely to be unable to get care.

In terms of mental effects of the pandemic and non-COVID-19 health status or habit, beneficiaries with forgone care tended to feel less financially secure and more lonely or sad and have depression. Forgone care was associated with e-cigarette usage and health conditions such as angina pectoris/coronary heart disease (CHD), congestive heart failure, other heart cond such as abnormal valve/rhythm, stroke/brain hemorrhage, cancer (non-skin), osteoporosis/soft bones, broken hip, emphysema/asthma/chronic obstructive pulmonary disease (COPD), weak immune system, and diabetes/high blood sugar. Alzheimers/dementia is negatively associated with self-reported forgone care.

#### 3.2.2. Telehealth coverage

Among the 43 variables, 39 were significantly associated with coverage of telehealth. The three most informative factors are Medicare-Medicaid dual eligibility (VIMP = 4.81, SE = 0.59 *p* < 0.001, OR = 0.58), residing area (metro residence, VIMP = 4.57, SE = 0.33, *p* < 0.001, OR = 1.87), and race/ethnicity (VIMP = 4.13, SE = 0.39 *p* < 0.001, OR = 2.00), indicating that non-dual-eligible beneficiaries (not eligible for Medicaid benefits), non-hispanic white and metro residents were more likely to have telehealth coverage provided by PCP.

The age group of 65–74 years old, the female gender group and the midwest/west-region group had higher coverage of telehealth. Beneficiaries using English language at home and those with higher income also had higher coverage. Owning a computer or smartphone with access to the Internet and usage of video/voice calls was positively associated with telehealth coverage. Respondents with telehealth coverage reported being able to pay rent/mortgage and get food, feeling more financially secure but more stressed, more lonely or sad, less socially connected, and having depression. Being able to get home supplies is negatively associated with telehealth coverage. Most variables describing non-COVID-19 health conditions and smoking status are negatively associated with the outcome, except cancer (non-skin), osteoporosis/soft bones/broken hip, and emphysema/asthma/COPD.

#### 3.2.3. Variable interactions

**Figure 3:**
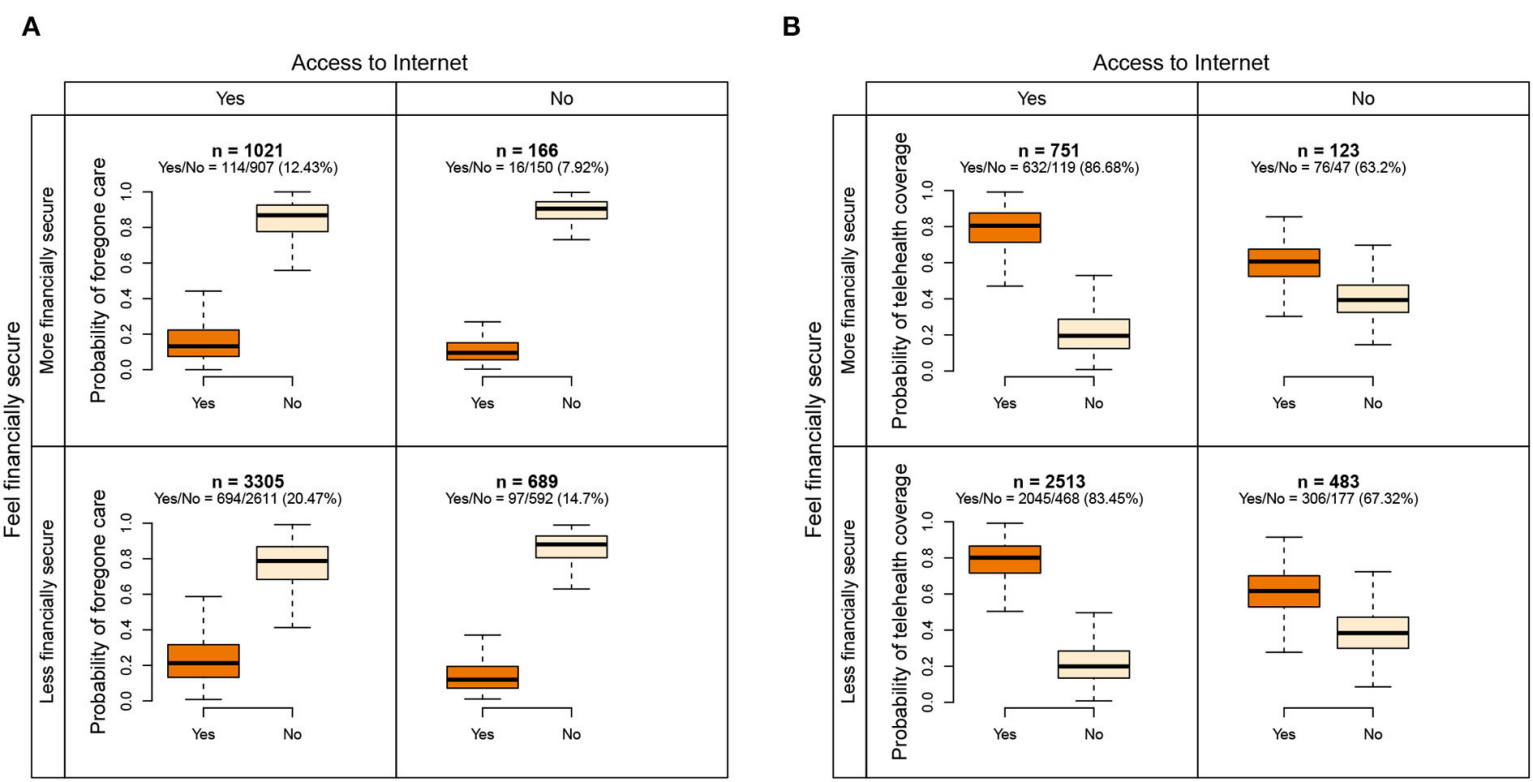
Interaction of access to the internet and the status of whether beneficiaries felt financially secure during the pandemic. The survey weighted proportions of positive outcomes are listed in parentheses. **(A)** The interaction for predicting forgone care. **(B)** The interaction for predicting telehealth coverage.

We detected three pairs of variables that intensified the disparity in both outcomes when different statuses were combined. Figures 3A,B demonstrate the interaction between statuses of Internet access and whether respondents felt financially secure during the pandemic. The group with Internet access and felt less financially secure had higher probabilities of forgone care (20.47%) than the group without Internet access and felt more financially secure (7.92%). The group with Internet access and felt more financially secure had higher probabilities of telehealth coverage (86.68%) than the group without Internet access and felt less financially secure (67.32%). Medicare-Medicaid dual eligibility interacted with the variable income, as shown in Supplementary Figures 2A,B. The higher-income group with full eligibility had higher probabilities of forgone care and telehealth coverage (17.88 and 84.98%) than the lower-income group not eligible for Medicaid (non-dual, 9.6 and 74.28%). The female group with the status of metro residence had higher probabilities of telehealth coverage (83.62%) than the male group with the status of non-metro residence (71.29%), as shown in Supplementary Figure 2D. However, such a difference is small for forgone care (12.26 vs. 10.08%), as shown in Supplementary Figure 2C, indicating that forgone care is not caused by telehealth coverage for this subgroup.

## 4. Discussion

Utilizing three waves of nationally representative survey data for Medicare beneficiaries, we analyzed trends in and reasons for forgone care and telehealth coverage during the COVID-19 pandemic. Although the percentage of forgone care decreased largely during the COVID-19 pandemic, telehealth coverage increased only on a small scale. Although CMS temporarily provided reimbursement for telehealth regardless of patient location (36), patient-based barriers to access to telehealth may still exist.

We found that the disparity in access to care through telehealth was associated with age, Medicare-Medicaid dual eligibility, electronic device usage, ability to access basic needs, and certain mental and physical health conditions. Among these factors, some socio-demographic factors had a similar influence in some prior studies reporting pre-COVID disparities in access to care (8, 37, 38); other factors may reflect an increased risk of spillover effects of the COVID-19 on non-COVID patients, including provision of essential chronic care (39) and changes in mental health (40). People with underlying chronic conditions are more susceptible to the infection due to weakened immunity, and therefore more likely to forgo needed treatments (41).

After adjusting for other factors, residence (metro or non-metro), region (northeast, midwest, south, or west) and income were not significantly associated with forgone care because of COVID-19, but they were significantly associated with telehealth coverage. As (42) stated, stated, health care partners should be informed about the collaborative use of telehealth-centered strategies to improve facility outcomes during the COVID-19 outbreaks. The disparity in telehealth coverage will eventually be reflected in poor access to care unless rapidly technological solutions are deployed and components of equity are examined. Further challenges replicating in-person care using telehealth formats include comorbidities. Take heart conditions, for example. Our data showed that Medicare beneficiaries with heart conditions were more likely to forgo care and less likely to have telehealth coverage. Radhakrishnan et al. (43) reported that for patients with heart failure on telehealth, comorbidity characteristics of renal failure, cancer, and depression comorbidities were significantly associated with withdrawal from telehealth services. However, a more recent study showed that for older persons living with HIV, the number of comorbidities was positively related to telehealth use *via* telehealth apps such as the MyChart App (44). Because of risk factors for severe COVID-19, the role of telehealth use will become more and more critical for the early identification of patients who need their care, care coordination, and the assessment of daily facility needs.

Medicare beneficiaries with depression had higher coverage of telehealth, indicating the absence of inequities between mental health coverage and coverage for other medical conditions. However, depression is positively associated with forgone care. This may reflect the fact that the share of adults with common mental disorders (primarily anxiety and depression), post-traumatic stress disorder, substance use disorders, behavioral disorders and suicidal behavior increased during the pandemic (45–47). Social isolation and lack of access to medical or behavioral health care may be associated with negative mental health outcomes (48). Therefore, it is important to consider how these factors are associated and explore ways to foster health system resilience to support vulnerable patients.

In contrast to previous studies (3, 18, 49), we analyzed related factors in a more inclusive fashion for ranking variables and identifying complex interactions. After adjustment for different factors, the discoveries could be more reproducible. For survey data with a large set of correlated variables, flexible statistical assumptions of the prediction model are often required. We are able to show that Random Forest provides a set of useful prediction tools when applied to a standard national survey data set. For this dataset, classical logistic regression and lasso penalized logistic regression suffered from multicollinearity and missing data problems, while Random Forest could provide prediction accuracy as high as almost 90%. Further, Random Forest provides an interpretable nonparametric variable important index that is useful for variable ranking (22, 50, 51). Although regular logistic regression suffers the problem of missing data, penalized logistic regression provides similar prediction performance. Overall, we saw potentially significant returns to statistical and machine learning methods.

### 4.1. Limitations

Because of the nature of survey data, this study is subject to recall and social desirability biases. Its results are not generalizable to non-medicare beneficiaries. In addition, we do not yet have data recording beneficiaries' education level or reasons for accessing telehealth. The variables we used were defined in wide categories with few details. For example, age was coded on three levels, income was recorded on only two levels, and measures of mental wellbeing were not sufficiently defined for different aspects. Finally, our findings should be interpreted cautiously because they were based on analyses addressing prediction or association, not causality.

## 5. Conclusions

In conclusion, existing barriers to telehealth may influence patients' forgone care during the COVID-19 pandemic. There is a need to develop telehealth services, enhance patients' awareness of telehealth, and ensure equal access and utilization of telehealth. Identifying the associations among forgone care, telehealth coverage and patients' socio-demographic and clinical characteristics is essential for policymakers, patients and clinics in making informed health care decisions.

## Data availability statement

The data are publicly available on GitHub: https://github.com/luminwin/MCBS_2020_2021.

## Author contributions

ML: conceptualization, methodology, software, formal analysis, investigation, writing—original draft preparation, writing—review and editing, supervision, and funding acquisition. XL: project administration and writing—review and editing. All authors have read and agreed to the published version of the manuscript.

## Funding

This study was funded by the Department of Public Health Sciences 2022 Copeland Foundation Award and the Relief Funding Award from the Office of the Vice Provost for Research and Scholarship and the Office of Faculty Affairs, University of Miami.

## Conflict of interest

The authors declare that the research was conducted in the absence of any commercial or financial relationships that could be construed as a potential conflict of interest.

## Publisher's note

All claims expressed in this article are solely those of the authors and do not necessarily represent those of their affiliated organizations, or those of the publisher, the editors and the reviewers. Any product that may be evaluated in this article, or claim that may be made by its manufacturer, is not guaranteed or endorsed by the publisher.

## References

[1] Hanna TP King WD Thibodeau S Jalink M Paulin GA Harvey-Jones E. Mortality due to cancer treatment delay: systematic review and meta-analysis. BMJ. (2020) 371:m4087. 10.1136/bmj.m4087 33148535 PMC7610021

[2] Clark CR Ommerborn MJ Coull B Pham DQ Haas JS. Income inequities and Medicaid expansion are related to racial and ethnic disparities in delayed or forgone care due to cost. Med Care. (2016) 54:555. 10.1097/MLR.0000000000000525 26974677 PMC5830941

[3] Park S Stimpson JP. Trends in self-reported forgone medical care among medicare beneficiaries during the COVID-19 pandemic. JAMA Health Forum. (2021) 2:e214299. 10.1001/jamahealthforum.2021.4299 35977302 PMC8796880

[4] Park C Ng BP Kim K. Inability to access health care due to COVID-19 among medicare beneficiaries. Am J Manag Care. (2022) 28:75–80. 10.37765/ajmc.2022.88823 35139292

[5] Busch AB Huskamp HA Raja P Rose S Mehrotra A. Disruptions in care for medicare beneficiaries with severe mental illness during the COVID-19 pandemic. JAMA Netw Open. (2022) 5:e2145677. 10.1001/jamanetworkopen.2021.45677 35089352 PMC8800078

[6] Whaley CM Pera MF Cantor J Chang J Velasco J Hagg HK. Changes in health services use among commercially insured US populations during the COVID-19 pandemic. JAMA Netw Open. (2020) 3:e2024984. 10.1001/jamanetworkopen.2020.24984 33151319 PMC7645698

[7] Reece JC Neal EF Nguyen P McIntosh JG Emery JD. Delayed or failure to follow-up abnormal breast cancer screening mammograms in primary care: a systematic review. BMC Cancer. (2021) 21:1–14. 10.1186/s12885-021-08100-3 33827476 PMC8028768

[8] Cantor JH McBain RK Pera MF Bravata DM Whaley CM. Who is (and is not) receiving telemedicine care during the COVID-19 pandemic. Am J Prevent Med. (2021) 61:434–8. 10.1016/j.amepre.2021.01.030 33781622 PMC7936544

[9] Garfan S Alamoodi AH Zaidan BB Al-Zobbi M Hamid RA Alwan JK. Telehealth utilization during the COVID-19 pandemic: a systematic review. Comput Biol Med. (2021) 138:104878. 10.1016/j.compbiomed.2021.104878 34592585 PMC8450049

[10] Hoffman DA. Increasing access to care: telehealth during COVID-19. J Law Biosci. (2020) 7:Lsaa043. 10.1093/jlb/lsaa043 32843985 PMC7337821

[11] Martinez-Martin N Dasgupta I Carter A Chandler JA Kellmeyer P Kreitmair K. Ethics of digital mental health during COVID-19: crisis and opportunities. JMIR Ment Health. (2020) 7:e23776. 10.2196/23776 33156811 PMC7758081

[12] Koonin LM Hoots B Tsang CA Leroy Z Farris K Jolly B. Trends in the use of telehealth during the emergence of the COVID-19 pandemic-United States, January-March 2020. Morbid Mortal Wkly Rep. (2020) 69:1595. 10.15585/mmwr.mm6943a3 33119561 PMC7641006

[13] Bose S Dun C Zhang GQ Walsh C Makary MA Hicks CW. Medicare beneficiaries in disadvantaged neighborhoods increased telemedicine use during the COVID-19 pandemic. Health Affairs. (2022) 41:635–42. 10.1377/hlthaff.2021.01706 35500186 PMC9843604

[14] Giacalone A Marin L Febbi M Franchi T Tovani-Palone MR. eHealth, telehealth, and telemedicine in the management of the COVID-19 pandemic and beyond: lessons learned and future perspectives. World J Clin Cases. (2022) 10:2363. 10.12998/wjcc.v10.i8.2363 35434056 PMC8968610

[15] Haroz EE Kemp CG O'Keefe VM Pocock K Wilson DR Christensen L. Nurturing innovation at the roots: the success of COVID-19 vaccination in American Indian and Alaska native communities. Am J Publ Health. (2022) 112:383–7. 10.2105/AJPH.2021.306635 35196058 PMC8887173

[16] Harris R Rosecrans A Zoltick M Willman C Saxton R Cotterell M. Utilizing telemedicine during COVID-19 pandemic for a low-threshold, street-based buprenorphine program. Drug Alcohol Depend. (2022) 230:109187. 10.1016/j.drugalcdep.2021.109187 34890927 PMC8619879

[17] Friedman AB Gervasi S Song H Bond AM Chen AT Bergman A. Telemedicine catches on: changes in the utilization of telemedicine services during the COVID-19 pandemic. Am J Manag Care. (2022) 28:e1–6. 10.37765/ajmc.2022.88771 35049260

[18] Ng BP Park C Silverman CL Eckhoff DO Guest JC Díaz DA. Accessibility and utilisation of telehealth services among older adults during COVID-19 pandemic in the United States. Health Soc Care Commun. (2022). 10.1111/hsc.13709 34994028

[19] Lu M Ishwaran H. Cure and death play a role in understanding dynamics for COVID-19: data-driven competing risk compartmental models, with and without vaccination. PLoS ONE. (2021) 16:e254397. 10.1371/journal.pone.0254397 34264960 PMC8282006

[20] Lu M. Dynamic modeling COVID-19 for comparing containment strategies in a pandemic scenario. Ann Biostat Biometr Appl. (2020) 4:1–4. 10.33552/ABBA.2020.04.00057932550241

[21] Breiman L. Random forests. Mach Learn. (2001) 45:5–32. 10.1023/A:1010933404324

[22] Ishwaran H Lu M. Standard errors and confidence intervals for variable importance in random forest regression, classification, and survival. Stat Med. (2019) 38:558–82. 10.1002/sim.7803 29869423 PMC6279615

[23] Ishwaran H Tang F Lu M Kogalur UB. RandomForestSRC: Multivariate Splitting Rule Vignette. (2021). Available online at: http://randomforestsrc.org/articles/mvsplit.html (accessed May 16, 2022).

[24] Lu M Parel JM Miller D. Interactions between staphylococcal enterotoxins A and D and superantigen-like proteins 1 and 5 for predicting methicillin and multidrug resistance profiles among *Staphylococcus aureus* ocular isolates. PLoS ONE. (2021) 16:e254519. 10.1371/journal.pone.0254519 34320020 PMC8318242

[25] Fang X Liu W Ai J He M Wu Y Shi Y. Forecasting incidence of infectious diarrhea using random forest in Jiangsu Province, China. BMC Infect Dis. (2020) 20:222. 10.1186/s12879-020-4930-2 32171261 PMC7071679

[26] Ong J Liu X Rajarethinam J Kok SY Liang S Tang CS. Mapping dengue risk in Singapore using random forest. PLoS Negl Trop Dis. (2018) 12:e0006587. 10.1371/journal.pntd.0006587 29912940 PMC6023234

[27] Coates-Brown R Moran JC Pongchaikul P Darby AC Horsburgh MJ. Comparative genomics of Staphylococcus reveals determinants of speciation and diversification of antimicrobial defense. Front Microbiol. (2018) 9:2753. 10.3389/fmicb.2018.02753 30510546 PMC6252332

[28] Lu M Sha Y Silva T Colaprico A Sun X Ban Y. LR hunting: a random forest based cell-cell interaction discovery method for single-cell gene expression data. Front Genet. (2021) 12:1431. 10.3389/fgene.2021.708835 34497635 PMC8420858

[29] Pasek J. With Some Assistance From Alex Tahk, Some Code Modified From R-Core; Additional Contributions by Gene Culter, Schwemmle M. Weights: Weighting Weighted Statistics. R package version 1.0.4. (2021). Available online at: https://CRAN.R-project.org/package=weights (accessed May 16, 2022).

[30] Ishwaran H Kogalur UB. Fast Unified Random Forests for Survival, Regression, Classification (RF-SRC). R package version 3.1.0 (2022). Available online at: https://cran.r-project.org/package=randomForestSRC (accessed May 16, 2022).

[31] Ishwaran H Lu M Kogalur UB. randomForestSRC: Variable Importance (VIMP) With Subsampling Inference Vignette (2021). Available online at: http://randomforestsrc.org/articles/vimp.html (accessed May 16, 2022).

[32] Hastie T Tibshirani R Friedman J. The Elements of Statistical Learning: Data Mining, Inference, and Prediction. New York, NY: Springer Science & Business Media (2009). 10.1007/978-0-387-84858-7

[33] Ishwaran H Lu M Kogalur UB. RandomForestSRC: Partial Plots Vignette (2021). Available online at: http://randomforestsrc.org/articles/partial.html (accessed May 16, 2022).

[34] Tibshirani R Bien J Friedman J Hastie T Simon N Taylor J. Strong rules for discarding predictors in lasso-type problems. J R Stat Soc Ser B. (2012) 74:245–66. 10.1111/j.1467-9868.2011.01004.x 25506256 PMC4262615

[35] Friedman J Hastie T Tibshirani R. Regularization paths for generalized linear models *via* coordinate descent. J Stat Softw. (2010) 33:1. 10.18637/jss.v033.i01 20808728 PMC2929880

[36] Hamadi HY Zhao M Haley DR Dunn A Paryani S Spaulding A. Medicare and telehealth: the impact of COVID-19 pandemic. J Eval Clin Pract. (2022) 28:43–8. 10.1111/jep.13634 34786796 PMC8657362

[37] Smith KT Monti D Mir N Peters E Tipirneni R Politi MC. Access is necessary but not sufficient: factors influencing delay and avoidance of health care services. MDM Policy Pract. (2018) 3:2381468318760298. 10.1177/2381468318760298 30288438 PMC6125037

[38] Fung V Graetz I Galbraith A Hamity C Huang J Vollmer WM. Financial barriers to care among low-income children with asthma: health care reform implications. JAMA Pediatr. (2014) 168:649–56. 10.1001/jamapediatrics.2014.79 24840805 PMC7105170

[39] Yoon S Goh H Chan A Malhotra R Visaria A Matchar D. Spillover effects of COVID-19 on essential chronic care and ways to foster health system resilience to support vulnerable non-COVID patients: a multistakeholder study. J Am Med Direc Assoc. (2022) 23:7–14. 10.1016/j.jamda.2021.11.004 34848198 PMC8585635

[40] Choi KR Heilemann MV Fauer A Mead M. A second pandemic: mental health spillover from the novel coronavirus (COVID-19). J Am Psychiatr Nurses Assoc. (2020) 26:340–3. 10.1177/1078390320919803 32340586

[41] Sanyaolu A Okorie C Marinkovic A Patidar R Younis K Desai P. Comorbidity and its impact on patients with COVID-19. SN Comprehens Clin Med. (2020) 2:1069–76. 10.1007/s42399-020-00363-4 32838147 PMC7314621

[42] Harris DA Archbald-Pannone L Kaur J Cattell-Gordon D Rheuban KS Ombres RL. Rapid telehealth-centered response to COVID-19 outbreaks in postacute and long-term care facilities. Telemed e-Health. (2021) 27:102–6. 10.1089/tmj.2020.0236 32644899 PMC7815058

[43] Radhakrishnan K Jacelon CS Bigelow C Roche JP Marquard JL Bowles KH. Association of comorbidities with home care service utilization of patients with heart failure while receiving telehealth. J Cardiovasc Nurs. (2013) 28:216–27. 10.1097/JCN.0b013e3182512331 22580628 PMC13011980

[44] Baim-Lance A Angulo M Chiasson MA Lekas HM Schenkel R Villarreal J. Challenges and opportunities of telehealth digital equity to manage HIV and comorbidities for older persons living with HIV in New York State. BMC Health Services Res. (2022) 22:5. 10.1186/s12913-022-08010-5 35524251 PMC9073813

[45] Pfefferbaum B North CS. Mental Health and the Covid-19 Pandemic. N Engl J Med. (2020) 383:510–2. 10.1056/NEJMp2008017 32283003

[46] Usher K Durkin J Bhullar N. The COVID-19 pandemic and mental health impacts. Int J Ment Health Nurs. (2020) 29:315–8. 10.1111/inm.12726 32277578 PMC7262128

[47] Kumar A Nayar KR. COVID 19 and its mental health consequences. J Mental Health. (2021) 30:1–2. 10.1080/09638237.2020.1757052 32339041

[48] Moreno C Wykes T Galderisi S Nordentoft M Crossley N Jones N. How mental health care should change as a consequence of the COVID-19 pandemic. Lancet Psychiatry. (2020) 7:813–24. 10.1016/S2215-0366(20)30307-2 32682460 PMC7365642

[49] Hsiao V Chandereng T Lankton RL Huebner JA Baltus JJ Flood GE. Disparities in telemedicine access: a cross-sectional study of a newly established infrastructure during the COVID-19 pandemic. Appl Clin Inform. (2021) 12:445–58. 10.1055/s-0041-1730026 34107542 PMC8189758

[50] Lu M Ishwaran H. A prediction-based alternative to P values in regression models. J Thorac Cardiovasc Surg. (2018) 155:1130. 10.1016/j.jtcvs.2017.08.056 29306487 PMC5915354

[51] Lu M Ishwaran H. Discussion on “nonparametric variable importance assessment using machine learning techniques” by Brian D. Williamson, Peter B. Gilbert, Marco Carone, and Noah Simon. Biometrics. (2021) 77:23–7. 10.1111/biom.13391 33290584 PMC7946645

