# Supplementary material for: Access to care through telehealth among U.S. Medicare beneficiaries in the wake of the COVID-19 pandemic
Source: Front Public Health. 2022 Sep 6;10:946944. doi: 10.3389/fpubh.2022.946944 (PMC9485666; doi:10.3389/fpubh.2022.946944)
Supplement: Supplementary file 1 [file Data_Sheet_1.PDF]

Supplementary Material for Access to Care Through Telehealth Among U.S. Medicare Beneficiaries in the Wake of the COVID-19 Pandemic

1 SUPPLEMENTARY TABLES

Table S1: Description of variables grouped by topic area.

	Number of Variables in Grouping		
Description	2020 Summer	2020 Fall	2021 Winter
Interview characteristics	3	3	3
Demographic information	9	10	11
Access to care during the pandemic	121	124	135
Personal experiences with COVID-19	45	42	42
Preventive measures and knowledge about COVID-19	27	43	61
Economic and mental effects of the pandemic	9	10	10
Non-COVID-19 health status	27	24	24

Table S2: Description of outcome variables.

Variable	Question*
Unable to get care because of COVID-19	Now I'd like to ask about care [you were/RESPONDENT (SP) was] unable to get because of the coronavirus pandemic.<br><br>Since (REFERENCE DATE), did [you/(SP)] need medical care for something other than coronavirus, but not get it because of the coronavirus pandemic?<br><br>[IF NEEDED: [Have you/Has (SP)] had any medical appointments rescheduled since (REFERENCE DATE) because of the coronavirus pandemic?<br>Or, [have you/has he/has she] needed a medical appointment but [were/was] unable to schedule one because of the coronavirus pandemic?]
Primary care physician (PCP) offers telehealth	Does [your/(SP)'s] usual provider offer telephone or video appointments, so that [you don't/he/she doesn't] need to physically visit their office or facility?<br><br>[IF NEEDED: Did [your/(SP)'s] provider offer to talk to [you/him/her] about [your/his/her] symptoms over the phone or video so that [you/he/she] wouldn't have to visit their office or facility?]<br><br>[IF NEEDED: Telephone appointments may include "audio-only" appointments.]

*The response was categorized as "yes", "no", "don't know", or "refused".

Table S3: Results from classification analysis using Random Forest (full version of Table 2).

	Unable to get care because of COVID-19				Primary care physician (PCP) offers telehealth				
Variable	Est	SE	P value	OR§	Est	SE	P value	OR	Sig‡
Age	0.13	0.04	0.002	1.08	0.49	0.10	0.000	0.68	**+++
Gender	-0.03	0.02	0.944	0.89	0.32	0.06	0.000	0.92	+++
Race/ethnicity group	0.11	0.12	0.169	1.43	4.13	0.39	0.000	2.00	+++
Metro residence	-0.03	0.05	0.716	1.05	4.57	0.33	0.000	1.87	+++
Region	0.12	0.07	0.057	1.08	1.10	0.15	0.000	0.97	+++
Income	-0.06	0.02	0.995	0.77	0.64	0.11	0.000	0.49	+++
Non-English	0.01	0.03	0.329	0.83	0.89	0.13	0.000	0.76	+++
Medicare-Medicaid dual eligibility	0.50	0.22	0.010	0.92	4.81	0.59	0.000	0.58	**+++
Own computer	-0.06	0.03	0.981	1.48	0.31	0.10	0.001	2.48	++
Own smartphone	-0.09	0.03	1.000	1.50	0.49	0.12	0.000	2.30	+++
Own tablet	0.00	0.02	0.525	1.38	0.02	0.06	0.392	2.03	
Access to Internet	-0.21	0.04	1.000	1.84	3.50	0.33	0.000	3.10	+++
Use video/voice calls	0.09	0.04	0.008	1.86	0.34	0.10	0.000	2.60	**+++
Able to pay rent/mortgage	0.41	0.18	0.013	0.61	1.40	0.35	0.000	1.20	*+++
Able to get food	0.90	0.40	0.012	0.39	2.17	0.45	0.000	1.32	*+++
Able to get home supplies	1.20	0.33	0.000	0.34	0.59	0.36	0.049	0.98	***+
Feel financially secure	0.71	0.15	0.000	0.56	1.49	0.27	0.000	1.21	***+++
Feel stressed	0.09	0.11	0.218	1.97	0.91	0.32	0.002	1.55	++
Feel lonely or sad	0.33	0.16	0.017	1.71	1.39	0.30	0.000	1.28	*+++
Feel socially connected	0.08	0.08	0.159	0.85	0.74	0.16	0.000	0.84	+++
Weak immune system (any reason)	0.39	0.06	0.000	1.89	-0.37	0.08	1.000	1.37	***
Hypertension/high BP	-0.03	0.02	0.884	0.97	0.32	0.06	0.000	0.87	+++
Myocardial infarction	-0.02	0.04	0.649	0.98	0.43	0.12	0.000	0.86	+++
Angina pectoris/CHD	0.23	0.09	0.005	1.38	0.63	0.16	0.000	0.98	**+++
Congestive heart failure	0.29	0.08	0.000	1.14	1.18	0.18	0.000	0.78	***+++
Other heart cond, eg valve/rhythm	0.04	0.02	0.035	1.22	0.29	0.07	0.000	0.91	*+++
Stroke/brain hemorrhage	0.29	0.06	0.000	1.07	0.50	0.16	0.001	0.81	***++
High cholesterol	0.00	0.02	0.495	1.09	0.27	0.06	0.000	0.97	+++
Cancer (non-skin)	0.06	0.03	0.031	1.16	0.42	0.08	0.000	1.06	*+++
Alzheimers/dementia	0.48	0.09	0.000	0.89	1.18	0.25	0.000	0.71	***+++
Depression	0.08	0.03	0.005	1.50	0.31	0.06	0.000	1.04	**+++
Osteoporosis/soft bones	0.10	0.03	0.000	1.37	0.19	0.06	0.000	1.09	***+++
Broken hip	0.22	0.12	0.037	1.00	0.89	0.31	0.002	0.86	*++
Emphysema/asthma/COPD	0.16	0.03	0.000	1.42	0.14	0.08	0.034	1.00	***+
Diabetes/high blood sugar	0.09	0.03	0.000	1.21	0.08	0.05	0.065	1.01	***
Any arthritis	-0.07	0.02	0.998	1.44	0.55	0.09	0.000	0.99	+++

Any heart condition	-0.01	0.02	0.791	1.20	0.05	0.04	0.138	0.87	
Any osteoporosis/broken hip	-0.02	0.02	0.802	1.30	0.16	0.05	0.001	1.07	++
Ever smoke cigarette/cigar/pipe	0.02	0.02	0.179	1.05	0.18	0.06	0.000	0.95	+++
Currently smoke cigarette/cigar/pipe	0.04	0.04	0.129	0.90	0.86	0.17	0.000	0.80	+++
Ever used e-cigarette	0.15	0.07	0.015	1.24	0.63	0.25	0.005	0.92	*++
Smoke e-cigarette now	0.06	0.15	0.344	1.12	3.70	0.33	0.000	0.86	+++
Interview date	2.09	0.27	0.000	-	1.16	0.42	0.003	-	***++

Est and SE indicate estimation and standard error for Random Forest variable importance (VIMP).

§ OR indicates survey-weighted odds ratio indicating the direction of effects: if the value is larger than one, the first category of the variable in Table 1 is more likely with a positive outcome than the second category. For example, the OR of age is 1.08, indicating that the 0 to 65 age group was more likely to forgo care than the 65 to 74 age group.

‡ Sig indicates significant level according to P values of VIMP: when the outcome is forgone care, * for $p \leq 0.05$, ** for $p \leq 0.01$, *** for $p \leq 0.001$; when the outcome is telehealth coverage, + for $p \leq 0.05$, ++ for $p \leq 0.01$, and +++ for $p \leq 0.001$.

Table S4: Results from classification analysis using penalized logistic regression.

	Unable to get care because of COVID-19		Primary care physician (PCP) offers telehealth	
	Coefficient†	Odds Ratio†	Coefficient	Odds Ratio
Intercept	-0.02	0.98	2.00	7.40
Age (65 - 74)	-0.19	0.83	0.23	1.26
Age (74+)	-	-	-0.16	0.85
Gender (Female)	0.28	1.32	-	-
Race/ethnicity group (Black non-Hispanic)	-	-	-0.40	0.67
Race/ethnicity group (Hispanic)	-0.16	0.85	-	-
Race/ethnicity group (Other/Unknown)	-0.17	0.84	-	-
Metro residence (Non-metro)	-	-	-	-
Region (Midwest)	0.16	1.18	-	-
Region (South)	-0.18	0.84	-0.48	0.62
Region (West)	0.28	1.33	-	-
Income ($25,000 or more)	-	-	-	-
Non-English (No)	-	-	-	-
Medicare-Medicaid dual eligibility (Nondual)	-	-	-	-
Medicare-Medicaid dual eligibility (Partial)	-0.30	0.74	0.14	1.15
Medicare-Medicaid dual eligibility (QMB only)	-	-	-	-
Own computer (No)	-	-	-0.51	0.60
Own smartphone (No)	-	-	-0.05	0.95
Own tablet (No)	-0.01	0.99	-	-
Access to Internet (No)	-	-	-	-
Use video/voice calls (No)	-0.42	0.66	-0.13	0.88

Able to pay rent/mortgage (Unable)	-	-	-	-
Able to pay rent/mortgage (Not needed)	-	-	-	-
Able to get food (Unable)	0.04	1.04	-	-
Able to get food (Not needed)	-0.25	0.78	0.32	1.37
Able to get home supplies (Unable)	0.36	1.43	-	-
Able to get home supplies (Not needed)	-0.11	0.89	-	-
Feel financially secure (Less secure)	0.41	1.51	-	-
Feel financially secure (About the same)	-	-	-	-
Feel stressed (Less stressed)	-0.38	0.68	-	-
Feel stressed (About the same)	-0.37	0.69	-0.30	0.74
Feel lonely or sad (Less lonely/sad)	-	-	-0.11	0.90
Feel lonely or sad (About the same)	-0.07	0.94	-	-
Feel socially connected (Less connected)	-	-	-	-
Feel socially connected (About the same)	-0.13	0.88	-	-
Weak immune system (any reason) (No)	-0.09	0.92	-0.14	0.87
Hypertension/high BP (No)	0.05	1.05	-0.01	0.99
Myocardial infarction (No)	-0.54	0.58	-	-
Angina pectoris/CHD (No)	-	-	-	-
Congestive heart failure (No)	-	-	-	-
Other heart cond, eg valve/rhythm (No)	-	-	-	-
Stroke/brain hemorrhage (No)	-	-	-	-
High cholesterol (No)	0.25	1.29	-	-
Cancer (non-skin) (No)	-0.17	0.84	-	-
Alzheimers/dementia (No)	-	-	-	-
Depression (No)	-	-	-	-
Osteoporosis/soft bones (No)	0.11	1.12	-0.07	0.93
Broken hip (No)	-	-	-	-
Emphysema/asthma/COPD (No)	-	-	-	-
Diabetes/high blood sugar (No)	-	-	-	-
Any arthritis (No)	-0.57	0.56	-	-
Any heart condition (No)	-	-	-	-
Any osteoporosis/broken hip (No)	-	-	-	-
Ever smoke cigarette/cigar/pipe (No)	-	-	-	-
Currently smoke cigarette/cigar/pipe (No)	-	-	-0.01	0.99
Ever used e-cigarette (No)	-	-	-	-
Smoke e-cigarette now (No)	-	-	-	-
Interview date (No)	-0.10	0.91	-	-

† Only non-zero coefficients are listed.

2 SUPPLEMENTARY FIGURES

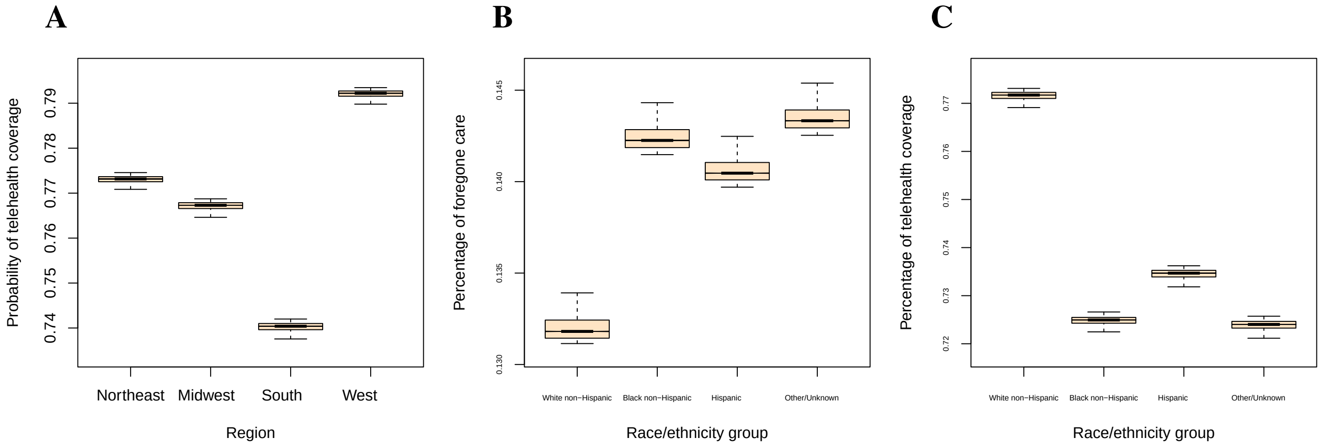

Figure S1: **Random Forest estimated probabilities of outcomes plotted against candidate variables after adjusting for other variables.** **(A)** The association between region and telehealth coverage. **(B)** The association between race/ethnicity and forgone care. **(C)** The association between race/ethnicity and telehealth coverage.

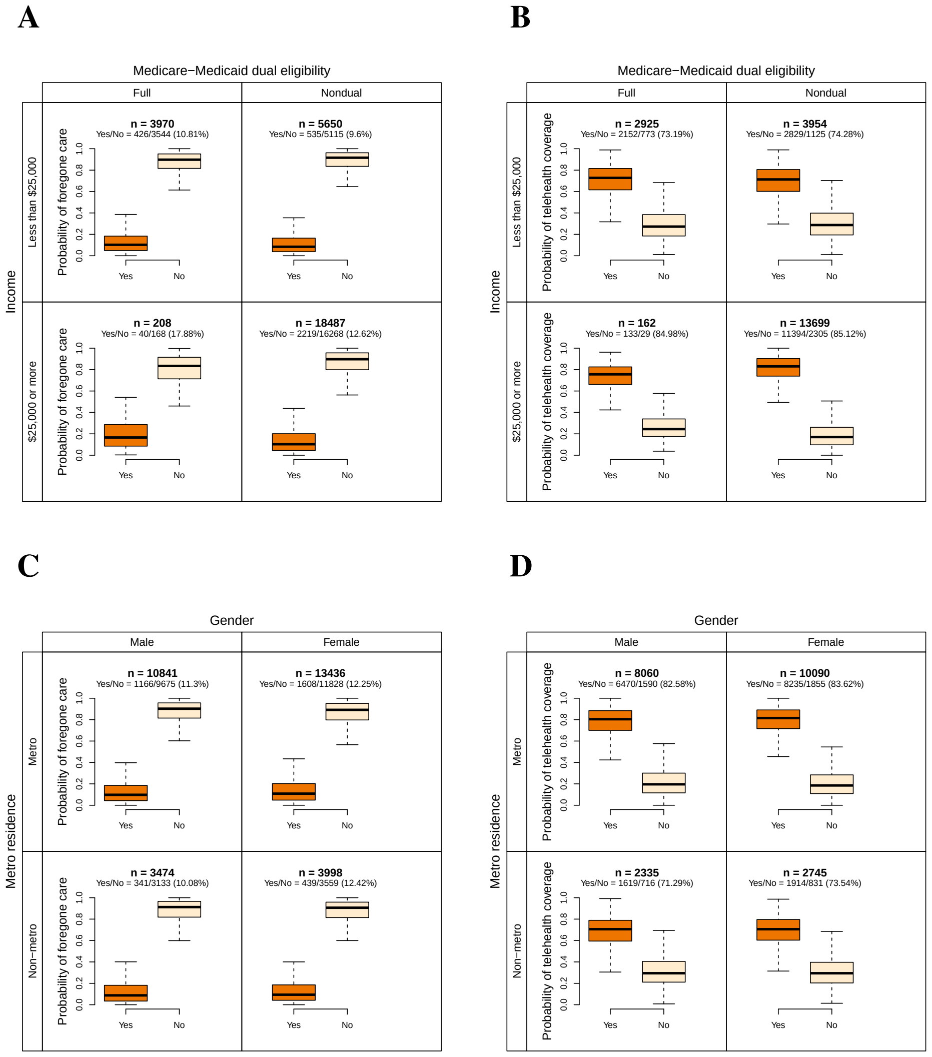

Figure S2: **Interactions of variables for predicting the probabilities of forgone care and telehealth coverage.** The survey-weighted proportions of positive outcomes are listed in parentheses. **(A)** The interaction between Medicare-Medicaid dual eligibility and income for predicting forgone care. **(B)** The interaction between Medicare-Medicaid dual eligibility and income for predicting telehealth coverage. **(C)** The interaction between gender and residing area for predicting forgone care. **(D)** The interaction between gender and residing area for predicting telehealth coverage.
